# Five-year patient-reported outcomes of individualized nutrition therapy supporting nutritional ketosis in adults with type 2 diabetes

**DOI:** 10.3389/fnut.2026.1855196

**Published:** 2026-08-14

**Authors:** Rebecca N. Adams, Shaminie J. Athinarayanan, Stephen D. Phinney, Jeff S. Volek, Amy L. McKenzie

**Affiliations:** 1 Virta Health Corp., Denver, CO, United States; 2 School of Medicine, University of California, Davis, Davis, CA, United States; 3 Department of Human Sciences, The Ohio State University, Columbus, OH, United States; 4 Lingo Biowearables, Alameda, CA, United States

**Keywords:** depression, knee pain, nutritional ketosis, sleep, type 2 diabetes

## Abstract

**Introduction:**

Sleep disturbance, depressive symptoms, and musculoskeletal pain are common in type 2 diabetes (T2D). The long-term patient-reported effects of nutrition-based interventions targeting metabolic dysfunction remain unclear. We examined 5-year changes in sleep, depressive symptoms, and knee pain in adults with T2D participating in a digitally delivered continuous care intervention providing individualized nutrition therapy supporting nutritional ketosis.

**Methods:**

Adults with T2D enrolled in the intervention arm of a non-randomized clinical trial were followed for 5 years (*N* = 194). Sleep was assessed using the Pittsburgh Sleep Quality Index (PSQI), depressive symptoms with the Center for Epidemiologic Studies Depression Scale (CES-D), and knee outcomes with the Knee Injury and Osteoarthritis Outcome Score (KOOS). Linear mixed-effects models evaluated change over time in the full cohort and symptom-defined subgroups. Clinical thresholds and minimal clinically important differences (MCID) were also examined.

**Results:**

PSQI scores improved at all follow-ups, with 19–25% reductions from baseline to 5 years; the proportion classified as poor sleepers decreased from 63 to 48%. Depressive symptoms decreased by 10 weeks and were sustained through 2 years; at 5 years the full cohort returned to baseline, but participants with baseline elevations maintained scores below the screening cut-off. KOOS subscales improved significantly; among those with baseline weekly knee pain, 57–74% exceeded the 10-point MCID.

**Discussion:**

A low-carbohydrate nutrition-focused, digital program targeting T2D was associated with durable sleep and pain benefits and sustained mood gains in symptomatic participants over 5 years.

## Introduction

Chronic diseases such as type 2 diabetes (T2D) are frequently accompanied by comorbid symptoms that extend beyond biomedical management. Depressive symptoms, musculoskeletal pain, and sleep difficulties occur at disproportionately high rates in T2D, in some cases two to three times higher than in the general population (1–3). These symptoms interact bidirectionally with metabolic health and with one another: depressive symptoms and poor sleep can worsen glycemic control, while hyperglycemia, systemic inflammation, excess adiposity, and medication burden contribute to the development or worsening of these conditions (4–12). These comorbidities represent clinically important targets in comprehensive T2D management.

Low carbohydrate nutrition has emerged as an effective therapeutic approach for T2D, producing improvements in glycemic control, body weight, and reduced need for diabetes medications (13). In our 2-year non-randomized controlled trial of a continuous remote care intervention (CCI) that emphasized nutritional ketosis through sustained carbohydrate reduction, participants achieved significant and sustained reductions in HbA1c, weight, and medication use compared with usual care (14). A 5-year extension study with the intervention arm confirmed durable improvements in glycemia, weight, and medication use (15). Beyond metabolic outcomes, we also reported improvements in depressive symptoms and knee pain (as a marker of musculoskeletal burden) at 2 years, and in sleep at 1 year (16–18). In a separate analysis from this cohort, we observed broad reductions in systemic inflammatory markers over 2 years (19), a finding consistent with hypothesized pathways linking nutritional ketosis to broader health outcomes (20–22). Together, these results provide the foundation for examining whether sustained carbohydrate reduction supporting nutritional ketosis is associated with durable patient-reported improvements over 5 years, a follow-up duration rarely reported in dietary intervention studies.

Reducing dietary carbohydrate intake may improve sleep difficulties, depressive symptoms, and knee pain by addressing these shared pathways. For example, weight loss can reduce mechanical load and inflammation contributing to musculoskeletal pain, and there is also preliminary evidence that very low carbohydrate diets may directly influence pain processing through metabolic and neurobiological mechanisms (5, 7, 23, 24). Emerging clinical evidence also suggests potential benefits of ketogenic dietary approaches for chronic pain, although available studies remain limited in duration and sample size (25). Improved glycemic control and reduced insulin resistance may also lessen metabolic stressors that disrupt sleep and mood, while reduced adiposity can alleviate obstructive sleep apnea (6, 10, 11, 21). Low carbohydrate nutrition has been associated with neutral to beneficial effects on mood and quality of life in clinical studies among individuals with T2D or obesity (26–29), and emerging work suggests ketogenic diets may hold promise for psychiatric disorders more broadly (30–33). Importantly, these symptoms also interact with one another: mood improvements may reduce pain perception and enhance sleep, while relief from pain or sleep disturbance may, in turn, improve mood (4, 8, 12). Taken together, these findings suggest that nutritional ketosis could influence both metabolic and psychosocial health. However, most studies of low-carbohydrate dietary strategies are limited to short- or medium-term follow-up, and long-term patient-reported outcomes remain poorly characterized. This gap provides a rationale for examining whether these effects persist over 5 years.

To address these gaps, we evaluated 5-year changes in sleep disturbance, depressive symptoms, and knee pain in adults with T2D participating in a CCI delivering individualized nutrition therapy supporting nutritional ketosis through lower carbohydrate intake. We examined outcomes in the overall sample and in subgroups with clinically significant baseline symptoms, and we assessed changes in the proportion exceeding clinical thresholds over time. This study extends our previously published findings on patient-reported outcomes at 1–2 years (16–18) and 5-year metabolic outcomes (15) by providing new evidence on the durability of improvements in mental and physical health within a digitally supported low-carbohydrate nutrition intervention.

## Materials and methods

### Participants and procedure

The detailed study design and primary outcomes were previously published (14, 15, 34), and the current results are based on a 5-year *post hoc* analysis using the data collected from the same cohort (Clinicaltrials.gov identifier: NCT02519309). Briefly, adults with T2D (ages 21–65 years and a body mass index of >25 kg/m^2^) initially enrolled in the intervention arm of a 2-year non-randomized, controlled trial were invited to continue for an additional 3 years of prospective follow-up. Of the 194 participants completing 2 years, 169 (87%) provided written informed consent for the extension and 122 (72.2%) were retained at 5 years. Participant flow through the 5-year extension, including extension consent and follow-up retention, is shown in Supplementary Figure 1.

Participants in the intervention arm were provided access to a web-based software application (app) that served as the central platform for care delivery. They were advised to achieve and maintain nutritional ketosis, defined as a blood β-hydroxybutyrate (BHB) level of 0.5–3.0 mmol/L, by maintaining carbohydrate intake below 30 g per day, with gradual adjustment based on individual tolerance and clinical goals. The application enabled two-way communication with a remote care team composed of a health coach and a medical provider. Through this system, participants received tailored guidance on nutrition, strategies for maintaining lifestyle changes, and medication management for diabetes and hypertension. They also uploaded body weight, blood glucose, and BHB values to the app, which were reviewed daily by the care team to deliver individualized feedback and recommendations.

The majority of the intervention was continuous and remote, but participants completed in-person study visits involving physical exam and laboratory measures at baseline, 10 weeks following dietary change, and 1-, 2-, 3.5, and 5 years following enrollment. Study questionnaires were completed either in-person (baseline through 2 years) or online (3.5 and 5 years).

### Measures

#### Baseline demographic and medical characteristics

Age, race, sex, and time since diabetes diagnosis were obtained from electronic medical records.

#### Sleep quality

Sleep quality was assessed with the 19-item Pittsburgh Sleep Quality Index (PSQI) (35). The PSQI contains seven component scores (including subjective sleep quality, sleep latency, sleep duration, sleep efficiency, sleep disturbance, use of sleep medication, and daytime dysfunction) and a global sleep score. The global PSQI score and three component scores—subjective sleep quality, sleep disturbances, and daytime dysfunction—were included in the current study, as these domains showed significant improvement at 1 year (18). The component scores range from 0 to 3 and the global score ranges from 0 to 21, with higher scores indicating poorer quality of sleep. PSQI is a widely-used measure of sleep quality and has strong reliability and validity evidence across clinical and non-clinical samples (36). In the current sample, internal consistency for the 19 items was good (ɑ = 0.70) at baseline. We also identified participants meeting the cut-off for “poor sleeper” (Global score >5) (35).

#### Depressive symptoms

Depressive symptoms were assessed with the 20-item Center for Epidemiological Studies Depression Scale [CES-D; (37)]. For continuous analyses, we summed 14 items for a total score based on prior research which supports the removal of 6 items (38, 39). The 14-item version of the CES-D has excellent reliability and validity evidence in the general population and among people with T2D. A sample item is “I felt that I could not shake off the blues even with help from my family and friends.” Responses range from 0 (Rarely or none of the time) to 3 (Most or all of the time). In the current sample, internal consistency for the 14 items was excellent (ɑ = 0.86) at baseline. We also identified participants meeting the clinical cut-off for depression using the full 20-item CES-D since the cut-off was established using that version; we used a clinical cut-off of 22 based on research showing that cut-off is most similar to established cut-offs on the PHQ-9 and Beck Depression Inventory (40) and a better indicator of Major Depressive Disorder in people with T2D (41) than the original CES-D cut-off of 16.

#### Knee pain

Knee pain was assessed with the Knee Injury and Osteoarthritis Outcome Score (KOOS) survey, a patient-reported outcome measure designed to assess the participant’s opinion about their knee and associated problems (42). The KOOS has five dimensions: Symptoms, Pain, Function in daily living (ADLs), Function in sport and recreation (Sports/Rec), and knee-related Quality of Life (QOL). Each subscale is scored separately on a scale from 0 to 100, where 0 indicates extreme knee problems and 100 represents no knee problems. The KOOS has strong evidence of reliability, validity, and sensitivity to change (43). In the current sample, internal consistencies for each of the subscales at baseline were: Symptoms = 0.90; Pain = 0.95; ADLs = 0.98; Sports/Rec = 0.97; QOL = 0.94. We also identified participants with baseline knee pain. In the absence of diagnostic information regarding knee osteoarthritis or an established clinical cut-off, responses to the question “How often do you experience knee pain?” were used to identify participants with knee pain at baseline; participants who reported at least “weekly” pain were considered to have knee pain.

### Statistical analyses

To assess changes over time in depressive symptoms, sleep quality, and knee pain, we used SPSS statistical software to perform linear mixed-effects models (LMMs) with time as a fixed effect and an unstructured covariance to account for within-person correlation; missing data were handled via maximum likelihood estimation under the assumption that data were missing at random. The number of participants contributing data for each patient-reported outcome at each follow-up time point is provided in Supplementary Table 1. We report the omnibus test of time from each LMM; to aid interpretation, the results also report baseline-referenced contrasts for each follow-up (two-sided *α* = 0.05). For sleep quality, the outcome variables were the Global Sleep score and 3 component scores: Sleep Quality, Sleep Disturbances, and Daytime Dysfunction. For depressive symptoms, the outcome variable was the 14-item version of the CES-D. For knee pain, the outcome variables were the Pain, Symptoms, ADLs, Sports/Rec, and QOL subscales. The covariates included in all models were baseline age, sex, race (African American vs. other), years since diabetes diagnosis, and baseline insulin use. Baseline antidepressant use was also included in models where the outcome variable was depressive symptoms. Each model was run twice: (1) with everyone who consented to the extension (“full sample”), and (2) only participants reporting relevant symptoms at baseline, i.e., met clinical cut-off for depression, were a “poor sleeper,” or endorsed weekly knee pain (“baseline symptom subgroup”).

**Table 2:** Estimated means (SE) from linear mixed-effects models for sleep, depression, and pain outcomes in the overall sample and in baseline-symptom subgroups.

Outcome	Sample	Baseline	10 wk	1 yr	2 yr	3.5 yr	5 yr	p for time effect
Global sleep	Overall sample	7.4 (0.28)	5.8 (0.25)***	6.0 (0.27)***	5.5 (0.26)***	6.1 (0.29)***	6.0 (0.28)***	<0.001
	Baseline poor sleepers	9.3 (0.28)	6.8 (0.32)***	6.9 (0.36)***	6.2 (0.34)***	7.2 (0.39)***	6.8 (0.33)***	<0.001
Sleep quality	Overall sample	1.1 (0.06)	0.8 (0.05)***	0.9 (0.06)***	0.8 (0.06)***	0.9 (0.06)***	0.9 (0.06)***	<0.001
	Baseline poor sleepers	1.4 (0.07)	1.0 (0.06)***	1.0 (0.07)***	0.8 (0.08)***	1.0 (0.09)***	1.0 (0.07)***	<0.001
Sleep disturbances	Overall sample	1.6 (0.05)	1.5 (0.04)**	1.3 (0.05)***	1.3 (0.04)***	1.3 (0.05)***	1.3 (0.05)***	<0.001
	Baseline poor sleepers	1.8 (0.06)	1.5 (0.05)***	1.4 (0.07)***	1.4 (0.06)***	1.4 (0.06)***	1.4 (0.06)***	<0.001
Daytime dysfunction	Overall sample	1.2 (0.06)	0.8 (0.06)***	0.7 (0.06)***	0.7 (0.06)***	0.8 (0.05)***	0.9 (0.06)***	<0.001
	Baseline poor sleepers	1.4 (0.07)	0.9 (0.09)***	0.8 (0.08)***	0.8 (0.08)***	1.0 (0.07)***	1.0 (0.08)***	<0.001
Depressive symptoms	Overall sample	8.6 (0.53)	6.4 (0.44)***	6.9 (0.54)**	7.2 (0.51)*	7.4 (0.67)	8.0 (0.61)	<0.001
	Baseline elevated depressive symptoms	23.3 (1.21)	13.8 (1.46)***	14.6 (1.93)***	20.5 (5.11)	16.8 (2.41)**	18.0 (2.12)*	<0.001
KOOS pain	Overall sample	81.9 (1.55)	85.2 (1.39)**	86.9 (1.33)***	87.8 (1.33)***	87.6 (1.23)***	89.6 (1.34)***	<0.001
	Baseline weekly knee pain	61.3 (2.52)	73.2 (2.54)***	75.9 (2.54)***	78.8 (2.29)***	78.1 (2.30)***	82.0 (3.05)***	<0.001
KOOS symptoms	Overall sample	79.3 (1.60)	82.6 (1.34)**	84.2 (1.26)***	84.8 (1.34)***	83.1 (1.42)*	86.0 (1.34)***	<0.001
	Baseline weekly knee pain	60.2 (2.80)	71.6 (2.28)***	73.8 (2.12)***	74.5 (2.22)***	74.9 (2.66)***	76.8 (2.87)***	<0.001
KOOS activities of daily living (ADL)	Overall sample	83.8 (1.45)	88.3 (1.27)***	88.9 (1.26)***	89.8 (1.28)***	90.6 (1.08)***	91.0 (1.27)***	<0.001
	Baseline weekly knee pain	67.5 (2.61)	78.9 (2.38)***	81.9 (2.09)***	82.5 (2.46)***	83.3 (2.32)***	84.9 (3.12)***	<0.001
KOOS sport and recreation function	Overall sample	69.9 (2.41)	75.8 (2.25)**	78.3 (2.18)***	79.9 (2.19)***	75.8 (2.29)**	76.9 (2.37)**	<0.001
	Baseline weekly knee pain	43.8 (3.86)	55.8 (4.39)**	62.1 (3.78)***	60.5 (4.29)***	55.1 (4.15)*	62.9 (4.66)**	<0.001
KOOS knee-related QOL	Overall sample	70.5 (2.19)	75.7 (2.13)***	78.8 (2.04)***	79.9 (1.94)***	78.3 (1.99)***	80.5 (1.95)***	<0.001
	Baseline weekly knee pain	42.7 (3.18)	54.5 (3.72)***	60.9 (3.64)***	64.0 (3.25)***	67.1 (3.28)***	72.0 (3.75)***	<0.001

Values are model-estimated means (SE) from linear mixed-effects models. Asterisks indicate significance for pairwise comparisons versus baseline (time 1), unadjusted for multiple comparisons: *p < 0.05, **p < 0.01, ***p < 0.001. Subgroup analyses were restricted to participants with clinically elevated symptoms at baseline, defined as CED-D ≥ 22 for depressive symptoms, PSQI global score > 5 for sleep disturbance, and self-reported weekly knee pain for KOOS outcomes. KOOS, Knee injury and Osteoarthritis Outcome Score; QOL, Quality of Life.

Additionally, we conducted analyses to assess the clinical significance of changes among participants with baseline and 5 year data. First, in the full sample, we examined changes in the percentage of participants meeting the clinical cut-off for depression or being a “poor sleeper” from baseline to 5 years using McNemar’s tests. We also planned to examine descriptive transitions in clinical status for these outcomes from baseline to 5 years (improved, stable, worsened). Among participants who endorsed weekly knee pain, we computed the percentage of participants who exceeded the minimal clinically important difference (MCID) of 10 for each subscale (44).

## Results

### Sample characteristics

At baseline, participants had a mean age of 54.2 ± 8.3 years, mean diabetes duration of 8.1 ± 6.9 years, BMI of 40.7 ± 8.5 kg/m^2^, and HbA1c 7.5 ± 1.4%. Participants were 65.1% female and 95.3% white (Table 1). Baseline characteristics did not differ between those who consented to the study extension and those who declined. Among participants enrolled in the extension, baseline characteristics also did not differ between those who completed 5 years of follow-up and those lost to follow-up. Participants with and without 5-year questionnaire data did not differ in baseline or 2-year patient-reported outcomes or baseline clinical characteristics.

**Table 1:** Baseline participant characteristics.

Characteristic	Value^1^
Demographics/medical	
Age, years	54.1 (8.3), 28.0–66.0
Female	110 (65.1%)
White	161 (95.3%)
Body Mass Index (BMI), kg/m^2^	40.7 (8.5), 24.8–86.9
Diabetes duration, years	8.1 (6.9), 0.0–33.0
Baseline insulin use	49 (29.0%)
Baseline antidepressant use	60 (35.5%)
Sleep	
PSQI global score	7.3 (3.6), 1.0–21.0
PSQI subjective sleep quality	1.1 (0.7)
PSQI sleep disturbances	1.6 (0.6)
PSQI daytime dysfunction	1.2 (0.7)
Poor sleepers (PSQI > 5)	96 (62.7%)
Depressive symptoms	
CES-D total score (14-item)	8.4 (7.4), 0.0–40.0
Meeting CES-D clinical cut-off (≥22, 20-item)	19 (12.3%)
Pain (KOOS)	
KOOS pain	82.7 (20.8), 13.9–100.0
KOOS symptoms	80.2 (21.4), 3.6–100.0
KOOS activities of daily living (ADL)	84.7 (19.8), 11.8–100.0
KOOS sport and recreation function	71.1 (33.1), 0.0–100.0
KOOS knee-related quality of life (QOL)	71.6 (30.2), 0.0–100.0
Weekly knee pain	54 (34.8%)

Values are M (SD), range for continuous variables and n (%) for categorical variables. Sample sizes vary slightly across measures due to missing data (sleep measures: *n* = 153–156; depressive symptoms: *n* = 155; KOOS subscales: *n* = 151–156). CES-D, Center for Epidemiologic Studies Depression Scale (14-item version); PSQI, Pittsburgh Sleep Quality Index; KOOS, Knee injury and Osteoarthritis Outcome Score.

At baseline, the mean PSQI global sleep score was 7.3, which exceeds the clinical cut-off of 5 for “poor sleep.” This suggests that participants, on average, reported impaired sleep, though mean scores were lower than those typically observed in clinical samples with diagnosed sleep disorders, where PSQI values often average 9–12 (35, 36). A total of 96 of 153 participants (62.7%) with available sleep data met the cut-off at baseline and were included in the subgroup analysis (Table 2).

The mean CES-D total score at baseline was 8.4 (14-item version), suggesting minimal to mild depressive symptoms on average. Nineteen of 155 participants (12.3%) with available depression data met the clinical cut-off (CES-D ≥ 22 on the 20-item version) and were included in the subgroup analyses (Table 2).

Baseline KOOS subscale scores ranged from 71 to 85, slightly lower (worse) than reference values from a general population sample (45). Fifty-four of 155 participants (34.8%) with available knee data endorsed at least weekly knee pain at baseline and were included in the subgroup analyses. In this subgroup, KOOS subscale scores ranged from 43 to 68, indicating substantially worse function compared with the general population (Table 2).

### Sleep

**Figure 1:**
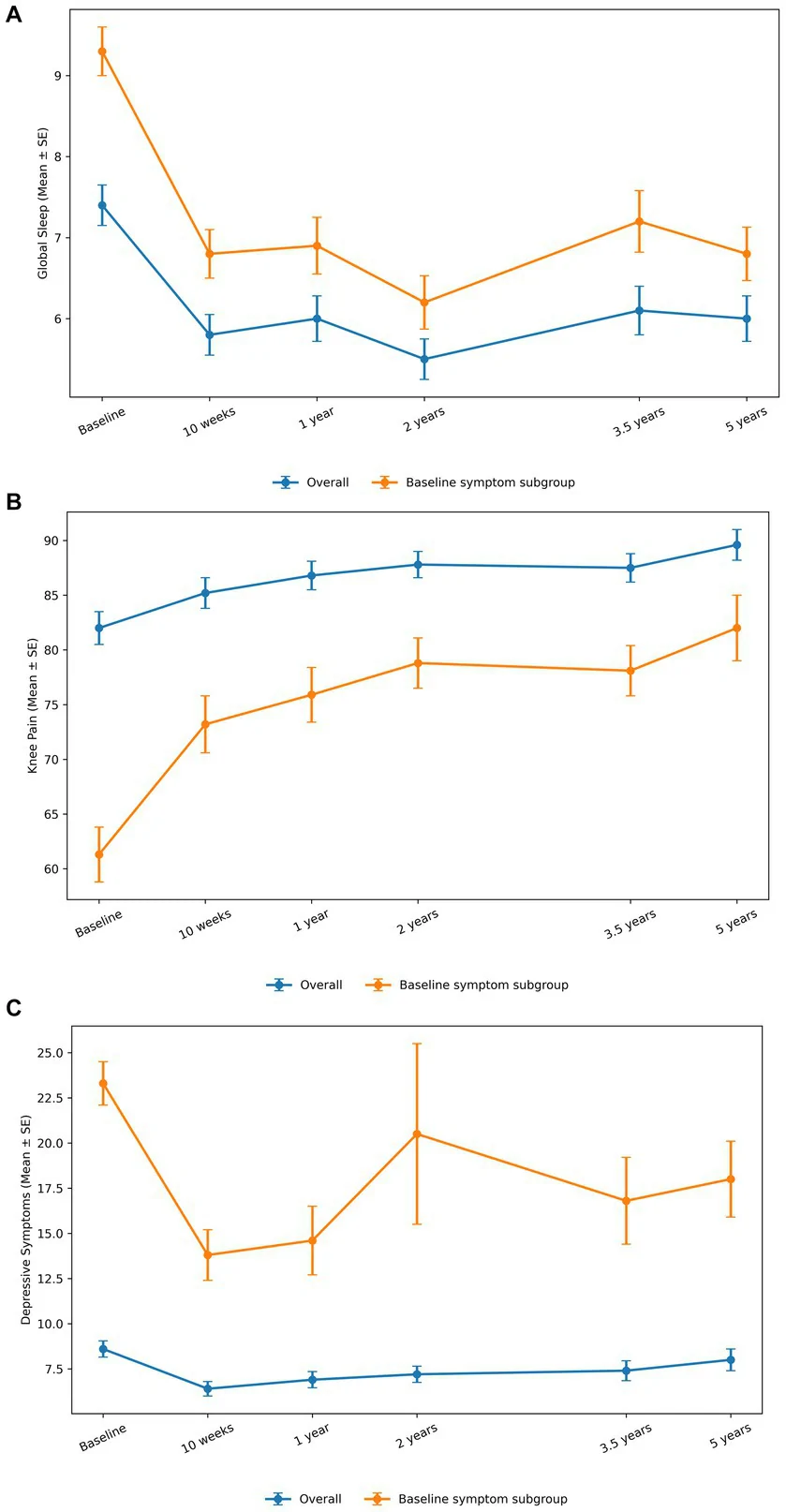
Longitudinal Trajectories of Patient-reported Outcomes Over 5 Years. **(A)** Global sleep (PSQI; overall N = 165, baseline poor sleeper subgroup n=93). **(B)** Depressive symptoms (CES-D–14; overall N = 165, baseline elevated symptoms subgroup n= 19). **(C)** Knee pain (KOOS Pain; overall N = 165, baseline knee pain subgroup n= 53). Lines show the overall cohort and the baseline symptom subgroup; points are model-estimated means with standard errors at baseline, 10 weeks, 1, 2, 3.5, and 5 years. Higher PSQI/CES-D = worse; higher KOOS = better. PSQI = Pittsburgh Sleep Quality Index; CES-D = Center for Epidemiologic Studies Depression Scale; KOOS = Knee injury and Osteoarthritis Outcome Score.

**Figure 2:**
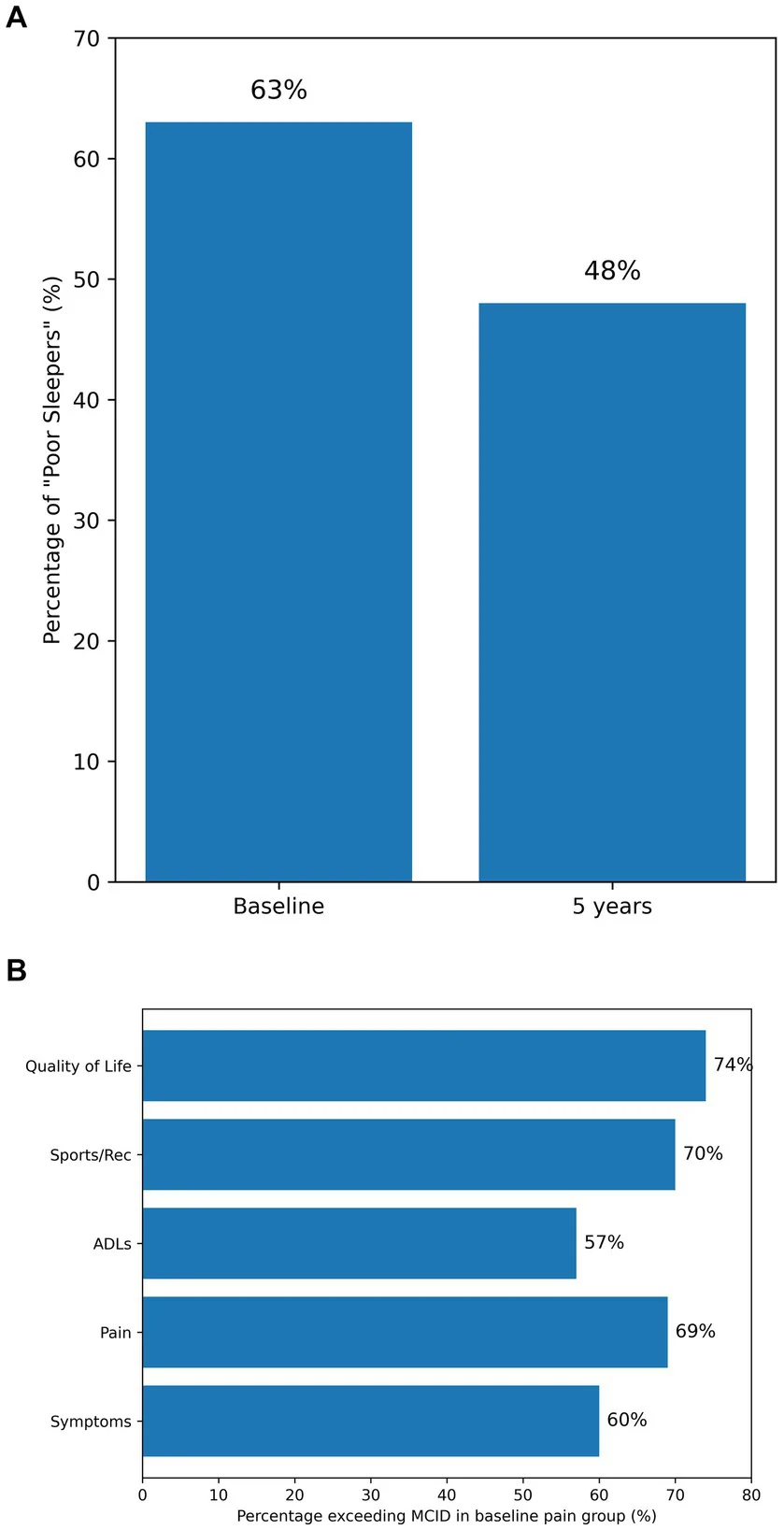
Clinical Significance at 5 Years. **(A)** Percentage classified as poor sleepers (PSQI > 5) at baseline vs. 5 years among paired completers (McNemar test; n = 105). **(B)** Percentage exceeding KOOS MCID (≥10 points) from baseline to 5 years for each subscale in the baseline weekly knee-pain subgroup (n = 35). MCID = minimal clinically important difference. ADLs = Activities of Daily Living; PSQI = Pittsburgh Sleep Quality Index; KOOS = Knee injury and Osteoarthritis Outcome Score; MCID = minimal clinically important difference.

There was a significant effect of time for PSQI Global and the three components in the overall cohort and among baseline poor sleepers (Table 2; Figure 1A). Compared with baseline, PSQI scores were significantly improved at each follow-up (all *p*s < 0.01). From baseline to 5 years, estimated mean reductions across PSQI outcomes ranged from 19 to 25%. Among participants with both baseline and 5-year data, the proportion classified as poor sleepers decreased from 63 to 48% (*p* = 0.01) (Figure 2A). For transitions in sleep status classification, 66% remained stable, 25% improved (no longer met the poor sleep cut-off), and 10% worsened (newly met the poor sleep cut-off).

### Depression

There was a significant effect of time for depressive symptoms in the overall cohort and among participants with clinically elevated depressive symptoms at baseline (Table 2; Figure 1B). In the overall sample, depressive symptoms improved significantly at 70 days, 1 year, and 2 years compared with baseline, but no differences were observed at 3.5 or 5 years (Figure 1B). Among participants with both baseline and 5-year data, the proportion meeting the CES-D clinical cut-off did not change significantly. Consistent with the non-significant change in prevalence, only a small number of participants shifted into or out of the elevated depressive symptom range over 5 years. In contrast, among participants with clinically elevated depressive symptoms at baseline (*n* = 19), depressive symptoms improved at 70 days, 1 year, 3.5 years, and 5 years, though not at 2 years.

### Knee pain

For all KOOS subscales, the effect of time was significant in the overall cohort and the baseline weekly knee-pain subgroup (Table 2). The KOOS Pain trajectory is shown in Figure 1C. Baseline-referenced contrasts were also significant at every follow-up (all *ps* < 0.05). Among participants with baseline knee pain, 60% exceeded the minimal clinically important difference (MCID; 10 points) for Symptoms, 69% for Pain, 57% for ADLs, 70% for Sports/Recreation, and 74% for Quality of Life (Figure 2B). These MCID analyses were limited to participants with both baseline and 5-year data.

## Discussion

In this 5-year extension of a CCI emphasizing nutritional ketosis, we observed sustained improvements in patient-reported sleep and knee pain, and clinically-meaningful benefit in depressive symptoms among those with baseline elevations. Few dietary intervention studies in T2D have reported patient-reported outcomes beyond 2–3 years, making the present 5-year findings notable in the context of sustained low carbohydrate nutrition supporting nutritional ketosis. Sleep quality improved in the overall cohort, with the proportion classified as poor sleepers declining significantly. Knee-related pain, function, and quality of life improved across all KOOS domains, with most participants surpassing the minimal clinically important difference. Depressive symptoms improved rapidly and were sustained through 2 years overall; by 5 years, average scores were no longer different from baseline in the full sample, but participants with baseline elevations maintained reductions below the clinical cut-off. These results extend our prior 1–2 year reports (16–18) and confirm that psychosocial benefits of carbohydrate reduction can persist alongside durable metabolic improvements (15). Notably, very few participants developed new sleep or depressive symptoms over 5 years, suggesting that improvements were accompanied by stability in those who began the intervention without clinically elevated concerns.

Sleep outcomes improved across the board, with reductions of roughly 20–25% in global and component scores. Importantly, the proportion classified as “poor sleepers” fell from nearly two-thirds at baseline to under half at 5 years. Such a shift represents a clinically meaningful change in population burden. Sleep improvements were moderate-to-large, comparable to effects reported in structured behavioral sleep interventions, and exceeded the modest magnitudes typically observed with pharmacotherapy (including non-benzodiazepine hypnotics and melatonin) (46–48). Consistent with our earlier findings in this cohort and with literature linking metabolic improvements and weight loss to better sleep, the durability at 5 years suggests that addressing metabolic dysfunction—through lower dietary carbohydrate intake, weight reduction, and improved glycemic stability—is associated with long-term improvements in subjective sleep (6, 10, 18).

Depressive symptoms improved within 10 weeks and were sustained through 2 years. By 5 years, mean scores in the overall cohort were no longer different from baseline; however, participants with clinically significant symptoms at baseline maintained reductions below the clinical screening cutoff. In the overall cohort, the proportion meeting the CES-D cutoff did not change significantly at 5 years, likely reflecting limited room to improve among participants without baseline elevations and heterogeneity in trajectories. In this context, durable benefit among those with baseline elevations is clinically meaningful given the high prevalence and clinical impact of depression in T2D (2, 11). Our findings build on prior 2-year results in this cohort (16) and a parallel randomized controlled trial (27) and extend follow-up to 5 years. The durability of benefit among those most symptomatic is especially encouraging, suggesting targeted real-world impact in a group often struggling with adherence and self-management. Improvements in depressive symptoms appeared to track with the metabolic gains previously reported in this cohort (15), consistent with evidence linking enhanced insulin sensitivity, reduced inflammation, and stabilized glycemia with improved mood regulation (22). They also align with emerging ketogenic research in mood disorders, where nutritional ketosis has been proposed and preliminarily shown to enhance brain energy metabolism, reduce neuroinflammation, and modulate neurotransmitter systems—collectively supporting improvements in mood and emotional regulation (30–33). Together, these findings underscore the interconnected nature of metabolic and psychological health and suggest that interventions improving metabolic flexibility may support durable well-being across both domains.

Knee-related outcomes improved robustly, with most participants exceeding the 10-point KOOS MCID across domains (~60–74% among those with baseline knee pain). Effect sizes from baseline to 5 years were small-to-moderate in the overall cohort and moderate-to-large among participants with baseline weekly knee pain, comparable to structured exercise therapy and on par with or exceeding many pharmacologic options that typically yield small-to-moderate short-term benefits (49, 50). Benefits are most plausibly explained by mechanical unloading from weight loss, with cohort-wide reductions in systemic inflammatory markers likely contributing (5, 7, 9, 19, 20, 22, 23, 51). These findings are also consistent with emerging literature suggesting that low-carbohydrate and ketogenic dietary approaches may influence chronic pain through metabolic and pain-processing pathways, although clinical evidence remains preliminary (23, 25). Clinically, less knee pain may enable greater physical function and self-care and may contribute to improvements in sleep and mood, reinforcing gains across domains (8, 12, 17).

These results suggest that sustained lower-carbohydrate nutrition may influence multiple interrelated patient-reported domains in T2D. In prior analyses from this cohort, early improvements in patient-reported outcomes were associated with or predicted by changes in metabolic and inflammatory markers, including reductions in central adiposity, body weight, C-reactive protein, insulin resistance (HOMA-IR), and higher blood β-hydroxybutyrate, a marker of nutritional ketosis (16–18). A single metabolic-focused program may be associated with broader and more durable gains than treating each symptom in isolation, with potential downstream benefits for adherence and quality of life.

Several considerations temper these findings but also point the way for future work. The parent trial’s initial 2-year phase was nonrandomized and the 5-year extension lacked a concurrent control arm; nonetheless, the ability to follow participants for 5 years within a digital continuous care model—while observing sustained changes—underscores the feasibility of long-term follow-up, even as causal inference across the full horizon remains limited. Because the intervention was delivered as a continuous remote care model, the relative contributions of dietary carbohydrate reduction and other aspects of the intervention, including individualized coaching and clinical support, cannot be separated. Patient-reported outcomes were secondary rather than primary endpoints, suggesting that future trials should be designed with these outcomes at the forefront. Baseline symptom subgroup analyses were also limited by modest sample sizes—most notably the depression subgroup (*n* = 19)—which constrains precision and increases the likelihood of unstable estimates. Therefore, findings from symptom-defined subgroups, particularly for depressive symptoms, should be interpreted cautiously. The larger standard errors and non-significant 2-year estimate within the depression subgroup despite improvement in the overall cohort further highlight the need for future studies to pre-specify and adequately power symptom-defined strata. Mechanistic assessments such as objective sleep measures, inflammatory biomarkers, and neurocognitive indices could clarify pathways linking nutritional ketosis to psychosocial change. Finally, our cohort was relatively homogenous; testing this model in more diverse populations and health system contexts will be critical for understanding generalizability. Together, these directions provide a roadmap for advancing long-term nutrition-based strategies supporting metabolic health in T2D.

## Conclusion

In conclusion, this 5-year extension of a digitally delivered, low-carbohydrate continuous remote care intervention was associated with durable improvements in sleep and knee pain and clinically meaningful, sustained reductions in depressive symptoms among participants with baseline elevations. Taken together, these outcomes suggest that sustained individualized nutrition therapy supporting nutritional ketosis can be associated with durable improvements in multiple patient-reported domains in adults with T2D over 5 years. These findings further support the potential role of metabolic-focused, digitally supported care as a pragmatic strategy for type 2 diabetes.

## Data Availability

The data analyzed in this study is subject to the following licenses/restrictions: the data presented in this study are available on request from the corresponding author. The data are not publicly available due to privacy and ethical restrictions. Requests to access these datasets should be directed to shaminie@virtahealth.com.
