# Supplementary material for: Five-year patient-reported outcomes of individualized nutrition therapy supporting nutritional ketosis in adults with type 2 diabetes
Source: Front Nutr. 2026 Aug 14;13:1855196. doi: 10.3389/fnut.2026.1855196 (PMC13521770; doi:10.3389/fnut.2026.1855196)

Supplementary Figure 1.

Participant Flow Diagram for the Continuous Care Intervention (CCI) Group, From Eligibility Screening Through 5-year Follow-up

Note. CCI = Continuous Care Intervention. Percentages reflect the proportion of enrolled participants (n = 262) retained at each follow-up time point. Participants who completed the original 2-year study were invited to provide written re-consent for a 3-year extension evaluating the effects of the CCI over 5 years of total follow-up.

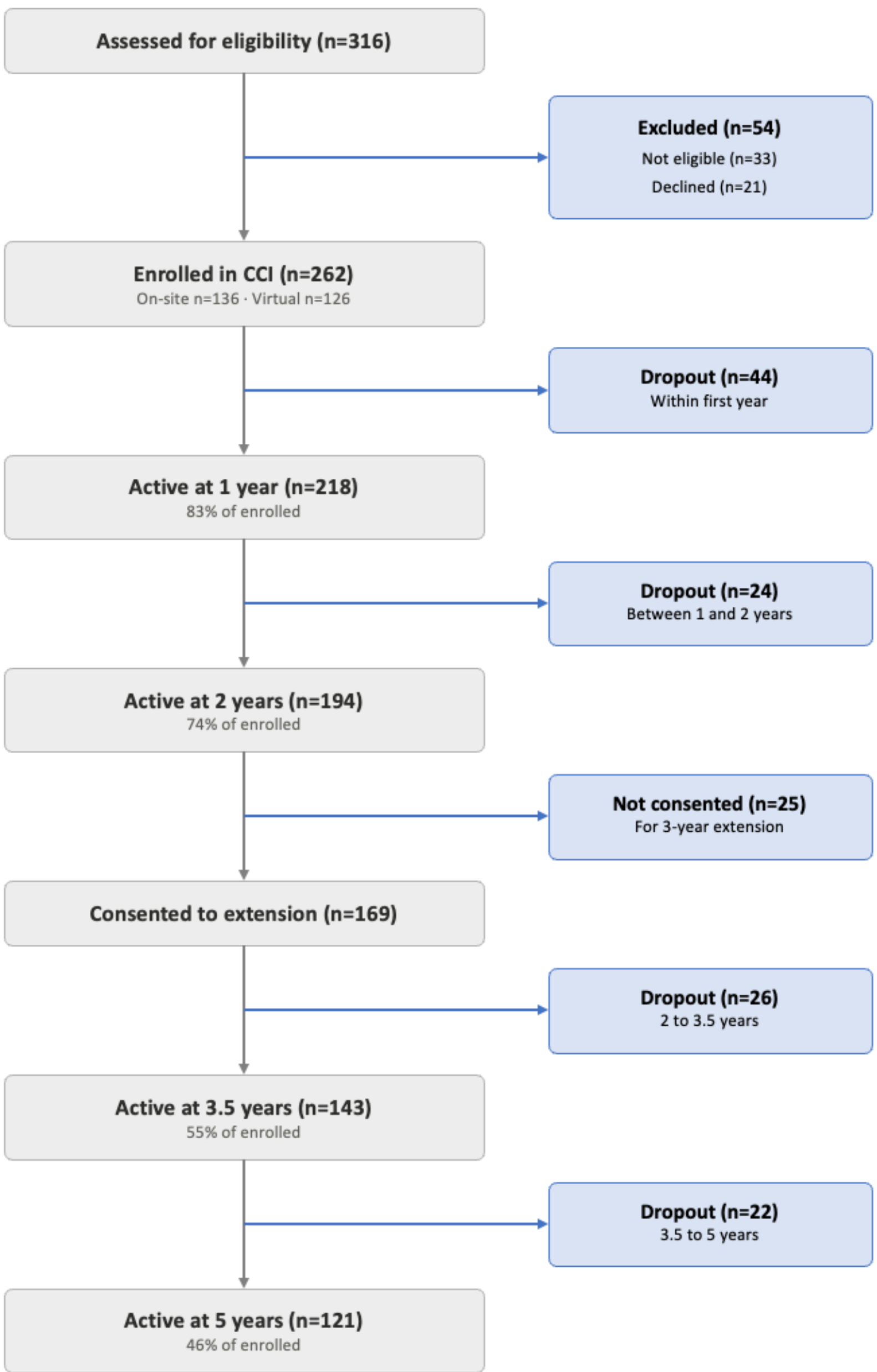

Supplement: Supplementary file 1 [file Image_1.pdf]
