# Supplementary material for: Five-year patient-reported outcomes of individualized nutrition therapy supporting nutritional ketosis in adults with type 2 diabetes
Source: Front Nutr. 2026 Aug 14;13:1855196. doi: 10.3389/fnut.2026.1855196 (PMC13521770; doi:10.3389/fnut.2026.1855196)
Supplement: Supplementary file 2 [file Table_1.docx]

**Supplemental Table 1**

*Number of Participants Contributing Patient-Reported Outcome Data at Each Follow-Up Time Point*

| **Outcome** | **Baseline** | | **10 weeks** | **1 year** | **2 years** | **3.5 years** | **5 years** |
| --- | --- | --- | --- | --- | --- | --- | --- |
| **Sleep (PSQI), all participants** |  | |  |  |  |  |  |
| Global Score | 153 | | 152 | 149 | 153 | 130 | 117 |
| Sleep Quality | 153 | | 153 | 151 | 151 | 130 | 117 |
| Sleep Disturbances | 155 | | 155 | 152 | 153 | 130 | 117 |
| Daytime Dysfunction | 156 | | 153 | 150 | 152 | 130 | 117 |
| **Sleep (PSQI), poor sleeper subgroup** | | |  |  |  |  |  |
| Global Score | | 96 | 84 | 85 | 88 | 74 | 66 |
| Sleep Quality | | 94 | 85 | 86 | 88 | 74 | 66 |
| Sleep Disturbances | | 96 | 86 | 86 | 88 | 74 | 66 |
| Daytime Dysfunction | | 96 | 85 | 85 | 88 | 74 | 66 |
| **Depression (CES-D), all participants** | | |  |  |  |  |  |
| CES-D Total Score | 155 | | 154 | 151 | 152 | 129 | 115 |
| **Depression (CES-D), baseline elevated depressive symptoms subgroup** | | | | | |  |  |
| CES-D Total Score | 19 | | 18 | 17 | 17 | 16 | 13 |
| **Knee symptoms (KOOS), all participants** | | | |  |  |  |  |
| Pain | 156 | | 154 | 149 | 153 | 129 | 117 |
| Symptoms | 156 | | 155 | 151 | 153 | 129 | 117 |
| Activities of Daily Living | 156 | | 154 | 151 | 153 | 129 | 117 |
| Sport/Rec | 151 | | 149 | 151 | 147 | 128 | 115 |
| Quality of Life | 153 | | 151 | 151 | 150 | 129 | 117 |
| **Knee symptoms (KOOS), baseline weekly knee pain subgroup** | | | | | |  |  |
| Pain | 54 | | 52 | 48 | 51 | 42 | 35 |
| Symptoms | 54 | | 52 | 50 | 51 | 42 | 35 |
| Activities of Daily Living | 54 | | 52 | 50 | 51 | 42 | 35 |
| Sport/Rec | 52 | | 50 | 50 | 50 | 41 | 35 |
| Quality of Life | 54 | | 51 | 50 | 51 | 42 | 35 |

**Note.** Participant numbers reflect observed questionnaire data available at each time point and may differ from the number of participants included in linear mixed-effects models, which used maximum likelihood estimation.
